# Intracranial Extramedullary Hematopoiesis Simulating a Subdural Hematoma: A Case Report

**DOI:** 10.7759/cureus.74206

**Published:** 2024-11-22

**Authors:** Wisam Al-Saad, Pir Abdul Ahad Aziz Qureshi, Vikram Rao Bollineni, Thordur Tryggvason

**Affiliations:** 1 Radiology, University Hospital Brussels, Brussels, BEL; 2 Faculty of Medicine, Háskóli Íslands, Reykjavík, ISL; 3 Radiology, Landspítali - The National University Hospital of Iceland, Reykjavík, ISL; 4 Pathology, Landspítali - The National University Hospital of Iceland, Reykjavík, ISL

**Keywords:** extramedullary hematopoeisis, intracranial bleed, intracranial extramedullary haematopoiesis, intracranial hemorrage, subdural hemorrhage

## Abstract

A 68-year-old patient came to the emergency department complaining of headaches and general weakness for the past month. The patient is known to have myeloproliferative disease. Non-contrast computer tomography showed a hyperdense extra-axial collection in bilateral frontoparietal regions, which was presumed to be bilateral subdural hematoma as the initial diagnosis. A drainage procedure using a borehole was performed, and a solid substance was aspirated instead of blood. Subsequently, the diagnosis of an extramedullary hematopoiesis was made using histopathological and immunohistochemical techniques.

## Introduction

Extramedullary hematopoiesis (EMH) refers to the production of blood cells outside of the bone marrow. This process occurs normally during fetal development when the bone marrow is still immature and can also occur pathologically when the bone marrow fails to produce stem cells properly [1]. It is a compensatory mechanism in response to chronic anemia secondary to hematopoietic disorders. The EMH is seen in patients with hemoglobinopathies such as sickle cell anemia, thalassemia, congenital spherocytosis, and myeloproliferative disorders, including polycythemia rubra vera and metaplastic myeloid leukemia [1]. Almost any organ or tissue can be affected by the process, including the gastrointestinal tract, intracranial structures, paraspinal region, adrenal glands, breast, thymus, kidneys, liver, spleen, and abdominal viscera. Intracranial extramedullary hematopoiesis (IEMH) is a rare presentation that can mimic metastasis, meningioma, and hematoma. 

## Case presentation

A 68-year-old patient presented to the emergency department with a one-month history of headache and general weakness. The patient had a known history of chronic myeloid leukemia, which was diagnosed in 2019. On general physician examination, the patient had a slight fever of 37.5°C. On neurological examination, cranial nerves 2 to 12 were normal; however, the patient had slight difficulty smiling; it appears bilaterally symmetrical. The patient also demonstrated symmetrical poor reflexes, negative pronator drift, and an inability to sit up unaided. The patient loses his balance when standing and needs support. The abdomen was soft and non-tender. There was hepatosplenomegaly. Bilateral lungs and cardiac auscultation were normal. Blood investigations were ordered, and the results are outlined in Table 1.

**Table 1:** Blood investigation results of the patient at the time of admission TLC: total leukocyte count; Hg: hemoglobin; CRP: C-reactive protein

Investigations	Values on admission	Reference range
TLC	6.9x10^9/L	4-10.5 x 10^9^/L
Hg	110 g/L	134-171 g/L
Platelets	49x10^9^/L	150-400 x 10^9^/L
CRP	21 mg/L	<10 mg/L
Blood glucose	10 mmol/L	4-6 mmol/L

**Figure 1:**
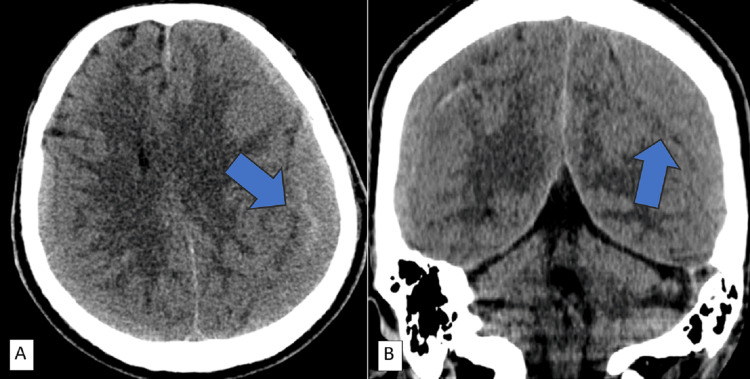
Non-contrast enhanced CT brain Axial (A) and coronal (B) views show bilateral subdural hyperdense collections in the frontoparietal regions (blue arrows).

A non-contrast CT revealed bilateral iso to slightly hyperdense extra-axial collections in bilateral frontoparietal regions with a density ranging between 45-50 Hounsfield units (HU) and a maximum width of 31 mm in the left superior parietal region. It caused a mass effect resulting in a midline shift of about 9 mm, which was interpreted as a bilateral subdural hematoma (Figure 1).

**Figure 2:**
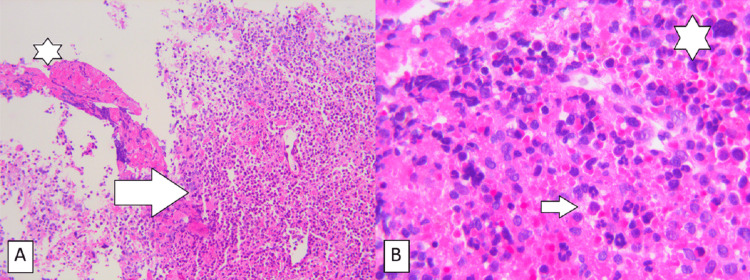
Histopathology of a sample taken from the subdural lesion (A) 100x magnification, H&E staining (A), showing extramedullary hematopoiesis (white arrow) around a connective tissue (asterisk), and (B) 400x magnification, H&E staining, showing hematopoietic cells. Megakaryocytes (asterisk), granulocytes, normoblasts, and plasma cells (arrow), vary in degree of maturation.

Subsequently, a drainage procedure using a burr hole was carried out, but no blood was evacuated; instead, a thick, solid substance of brown color was unexpectedly obtained. Samples were sent for histopathological and immunohistochemical examination, which showed a morphology consistent with hypercellular hematopoietic reactive bone marrow, a significant increase in megakaryocytes, myeloid precursors, and a large number of neutrophils. Fragments of bony tissue were also present. Further immunohistochemical study confirmed the diagnosis of extramedullary hematopoiesis (Figure 2).

**Figure 3:**
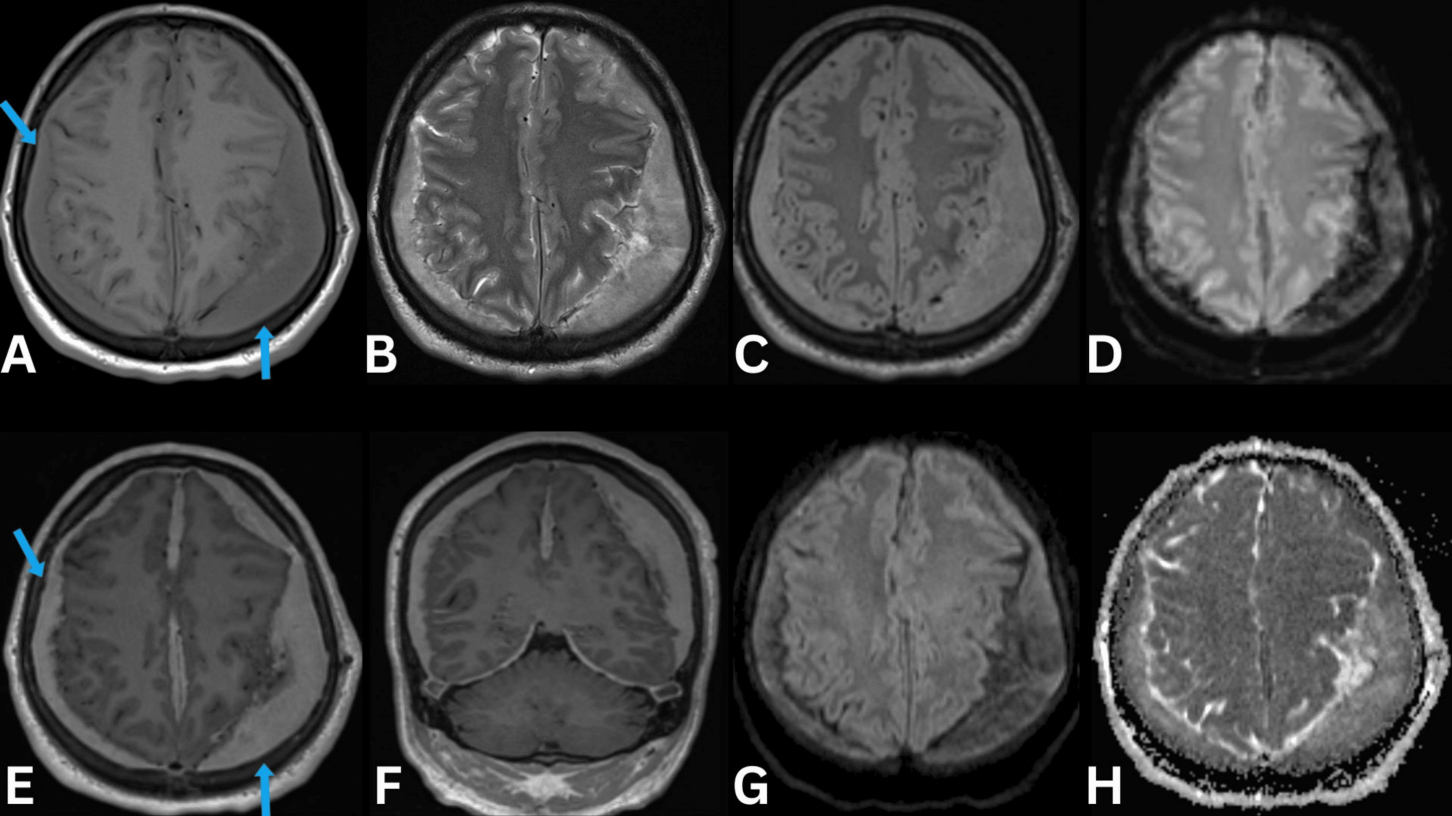
MRI brain with and without contrast T1-weighted axial view (A), T2-weighted axial view (B), FLAIR axial view (C), DWI axial view (D), T1-weighted post-contrast axial view (E), T1-weighted post-contrast coronal view (F), SWI axial view (G), and ADC axial view (H) reveal extra-axial lesions bilaterally along the cerebral convexities showing uniform mild post-contrast enhancement (blue arrows). These show iso to slightly hypointense T1 and heterogeneous T2 signal intensity, relatively high signal intensity on the FLAIR image, and susceptibility artifact on the SWI image, suggesting hemosiderin disposition. There is no diffuse restriction within the lesions. DWI: diffusion-weighted imaging; SWI: susceptibility-weighted imaging; ADC: apparent diffusion coefficient; FLAIR: fluid-attenuated inversion recovery

Furthermore, an MRI of the brain with and without contrast was also obtained after the burr hole procedure to further investigate the changes seen on the CT scan (while awaiting the pathological findings), which revealed mildly enhancing bilateral extra-axial lesions in frontoparietal regions (Figure 3).

## Discussion

EMH is a compensatory mechanism that occurs secondary to the inadequate production of blood cells by the bone marrow, often due to inherited blood disorders such as thalassemia, sickle cell anemia, or myeloproliferative disorders [1,2]. In children, IEMH is frequently linked with thalassemia, while in adults, it is often associated with myeloproliferative disease. Extramedullary hematopoiesis typically involves the reticuloendothelial systems, including lymph nodes, spleen, and mediastinum, but it can occur in almost any tissue in the body [3]. While para-vertebral EMH is well recognized, IEMH is extremely rare, with few cases reported. The mesenchymal origin of pluripotent stem cells in the central nervous system may explain its occurrence [4].

Historically, the first reported case of IEMH was observed in 1966, involving a severely anemic infant with a persistent subdural hematoma [5]. This case provided foundational insights into the potential mechanisms of IEMH and raised questions about whether it was a congenital anomaly or a compensatory response to anemia. Various theories attempt to explain this occurrence. Various theories attempt to explain this occurrence. One theory suggests that EMH is a compensatory phenomenon due to inadequate bone marrow space secondary to myelofibrosis or bone marrow metastases [6]. Koch et al. hypothesized “redirected differentiation theory,” which proposes that stem cells from various tissue types might transform into hematopoietic stem cells when stimulated by unidentified circulating factors in reaction to anemia or other blood-related disorders [6]. One case report also suggested that EMH can also occur in post-traumatic patients [5]. This occurrence has been noted in lung tissue after bone fractures or cardiac surgical procedures, as well as in the presacral region following a sacral fracture [7]. EMH in such cases is thought to occur secondary to the bone marrow embolism [5]. Li et al. presented one such case in which a patient without underlying hematological disorder developed EMH in the subdural hematoma secondary to a head injury and a skull fracture [5]. However, it is also worth mentioning here that subdural hematoma can also result from the EMH [5]. Nichols et al. presented EMH in a known beta-thalassemia patient who presented with a head injury [8]. 

In our case, the patient had a known underlying chronic myeloid leukemia and presented with a history of headaches and generalized weakness and was found to have extra-axial masses on further radiological investigations. The patient´s known status of hematological disorder in our case is also in line with many reported cases in the literature [9]. IEMH could be the initial presentation of the myeloproliferative disorder [10]. However, some cases of IEMH remain silent until the patient develops signs and symptoms of increased intracranial pressure, such as headaches.

On CT, IEMH typically appears as a hyperdense, extra-axial lesion, often mimicking subdural hematoma or tumors such as meningioma. Magnetic resonance imaging (MRI) is considered the imaging modality of choice [3]. On MRI, these lesions characteristically appear as well-defined lobulated single or multiple extra-axial masses. These lesions typically show intermediate signals on T1 and hypointense signals on T2-weighted images. On post-contrast images, these lesions show uniform post-contrast enhancement [3]. However, in our case, the lesion appeared relatively hyperintense on T2-weighted images, presenting a diagnostic challenge.

## Conclusions

IEMH should be considered in the differential diagnosis of intracranial masses in patients with myeloproliferative disease, even when imaging findings are atypical. Although IEMH usually involves the reticuloendothelial system, it can occur in any tissue, including the central nervous system, as seen in our patient. Therefore, recognizing this rare entity is crucial for accurate diagnosis and appropriate management, ultimately improving outcomes in patients with complex hematologic conditions.
